# Implicating type 2 diabetes effector genes in relevant metabolic cellular models using promoter-focused Capture-C

**DOI:** 10.1007/s00125-024-06261-x

**Published:** 2024-09-06

**Authors:** Nicholas A. Wachowski, James A. Pippin, Keith Boehm, Sumei Lu, Michelle E. Leonard, Elisabetta Manduchi, Ursula W. Parlin, Martin Wabitsch, Alessandra Chesi, Andrew D. Wells, Struan F. A. Grant, Matthew C. Pahl

**Affiliations:** 1 Center for Spatial and Functional Genomics, The Children’s Hospital of Philadelphia, Philadelphia, PA USA; 2 Division of Human Genetics, The Children’s Hospital of Philadelphia, Philadelphia, PA USA; 3 Department of Genetics, Perelman School of Medicine, University of Pennsylvania, Philadelphia, PA USA; 4 Department of Pediatrics, University of Pittsburgh School of Medicine, Pittsburgh, PA USA; 5 Division of Pediatric Endocrinology and Diabetes, Department of Pediatrics and Adolescent Medicine, University Medical Center Ulm, Ulm, Germany; 6 Department of Pathology and Laboratory Medicine, Perelman School of Medicine, University of Pennsylvania, Philadelphia, PA USA; 7 Department of Pathology, The Children’s Hospital of Philadelphia, Philadelphia, PA USA; 8 Institute for Immunology, Perelman School of Medicine, University of Pennsylvania, Philadelphia, PA USA; 9 Department of Pediatrics, Perelman School of Medicine, University of Pennsylvania, Philadelphia, PA USA; 10 Division of Diabetes and Endocrinology, The Children’s Hospital of Philadelphia, Philadelphia, PA USA

**Keywords:** Chromatin conformation, Epigenetics, Insulin secretion, SMCO4, Type 2 diabetes, Variant to gene mapping

## Abstract

**Aims/hypothesis:**

Genome-wide association studies (GWAS) have identified hundreds of type 2 diabetes loci, with the vast majority of signals located in non-coding regions; as a consequence, it remains largely unclear which ‘effector’ genes these variants influence. Determining these effector genes has been hampered by the relatively challenging cellular settings in which they are hypothesised to confer their effects.

**Methods:**

To implicate such effector genes, we elected to generate and integrate high-resolution promoter-focused Capture-C, assay for transposase-accessible chromatin with sequencing (ATAC-seq) and RNA-seq datasets to characterise chromatin and expression profiles in multiple cell lines relevant to type 2 diabetes for subsequent functional follow-up analyses: EndoC-BH1 (pancreatic beta cell), HepG2 (hepatocyte) and Simpson–Golabi–Behmel syndrome (SGBS; adipocyte).

**Results:**

The subsequent variant-to-gene analysis implicated 810 candidate effector genes at 370 type 2 diabetes risk loci. Using partitioned linkage disequilibrium score regression, we observed enrichment for type 2 diabetes and fasting glucose GWAS loci in promoter-connected putative *cis*-regulatory elements in EndoC-BH1 cells as well as fasting insulin GWAS loci in SGBS cells. Moreover, as a proof of principle, when we knocked down expression of the *SMCO4* gene in EndoC-BH1 cells, we observed a statistically significant increase in insulin secretion.

**Conclusions/interpretation:**

These results provide a resource for comparing tissue-specific data in tractable cellular models as opposed to relatively challenging primary cell settings.

**Data availability:**

Raw and processed next-generation sequencing data for EndoC-BH1, HepG2, SGBS_undiff and SGBS_diff cells are deposited in GEO under the Superseries accession GSE262484. Promoter-focused Capture-C data are deposited under accession GSE262496. Hi-C data are deposited under accession GSE262481. Bulk ATAC-seq data are deposited under accession GSE262479. Bulk RNA-seq data are deposited under accession GSE262480.

**Graphical Abstract:**

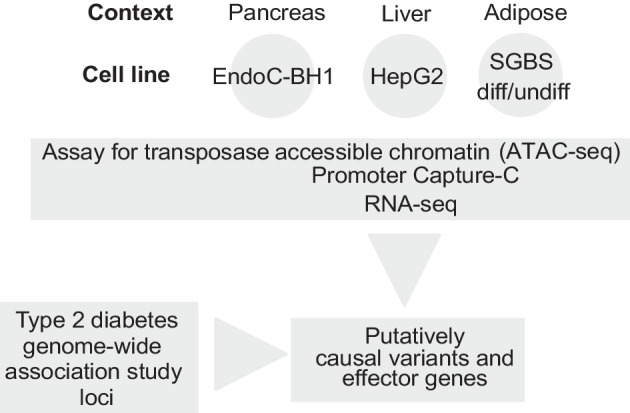

**Supplementary Information:**

The online version of this article (10.1007/s00125-024-06261-x) contains peer-reviewed but unedited supplementary material.


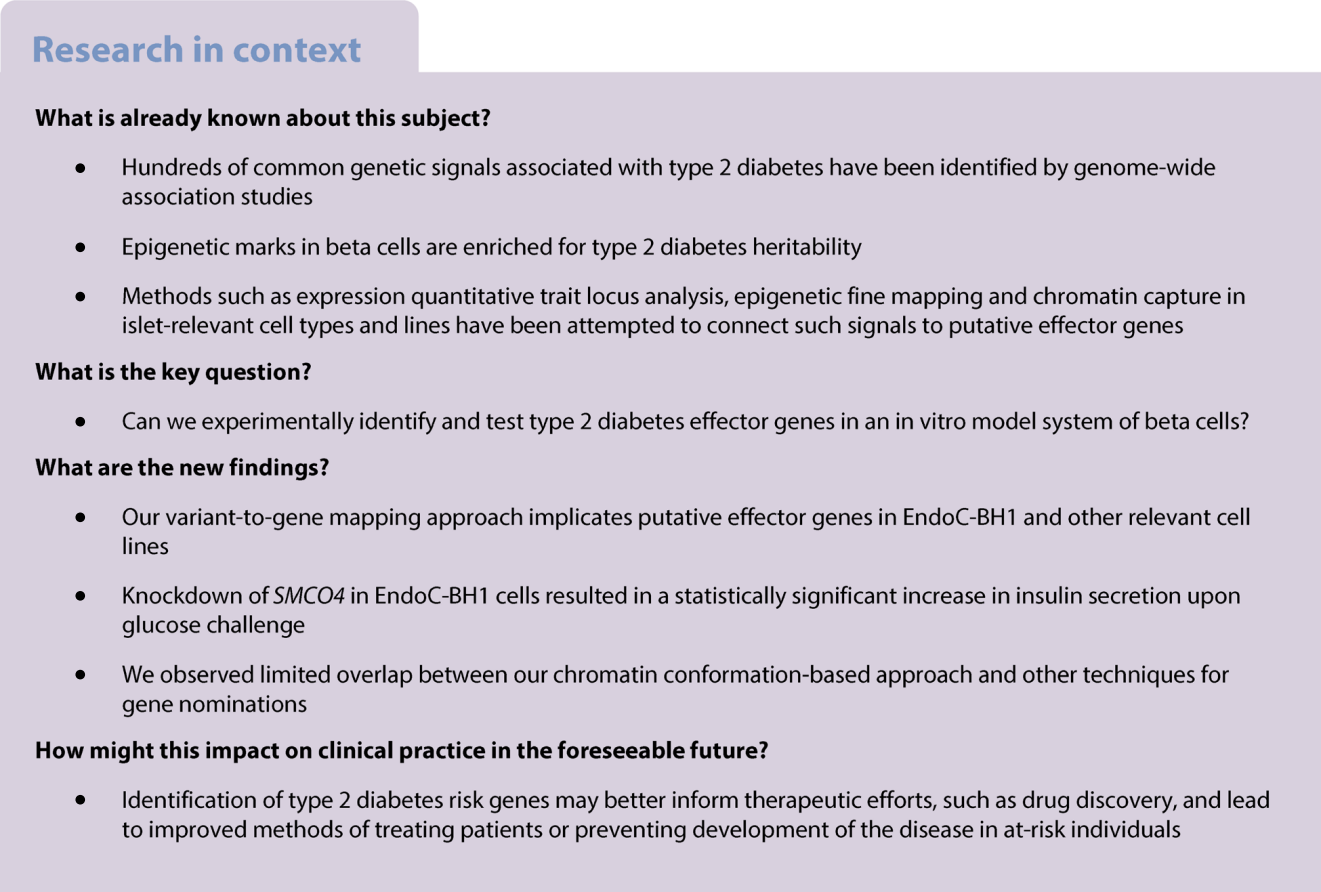


## Introduction

Genome-wide association studies (GWAS) have identified hundreds of common genetic variants robustly associated with increased susceptibility to type 2 diabetes. The overwhelming majority of these GWAS signals are located in non-coding regions and are thought to act by altering the activity of *cis*-regulatory elements (cREs) and subsequently affecting gene expression either locally or through distant chromatin contacts [1, 2].

There are several key challenges in elucidating the mechanism by which disease-associated variants contribute to type 2 diabetes risk. The GWAS approach does not unambiguously identify causal variants, i.e. the SNP(s) at a given locus molecularly responsible for the disease-associated risk. This is due to the fact that GWAS signals are typically in linkage disequilibrium (LD) with multiple proxy SNPs, any of which could be the causal variant(s). Additionally, GWAS does not identify the cellular context in which the variant confers its effect. While many type 2 diabetes variants are thought to act principally via pancreatic islets, other metabolic tissues such as liver and adipose are also considered critical in the disease aetiology. Finally, GWAS results in themselves do not connect putatively causal non-coding variants to their corresponding effector genes.

Given that GWAS-implicated SNPs do not always act via the nearest gene, mapping the epigenomic landscape in relevant cell types and cellular models presents an opportunity to improve causal variant nominations and gene selection for downstream functional studies [3, 4]. To this end, methods such as expression quantitative trait locus (eQTL) analysis, chromatin conformation/state profiling and RNA interference (RNAi)/CRISPR screening approaches have yielded a degree of success in identifying putative effector genes at type 2 diabetes GWAS loci [3, 5–9].

EndoC-BH1 cells have been shown to be a particularly powerful model for pancreatic beta cells, by recapitulating insulin secretion in response to glucose and sharing similar regulatory elements to human islets [6, 10]. Simpson–Golabi–Behmel syndrome (SGBS) cells provide an equally useful model that recapitulates adipocyte biology [11], while HepG2 cells are a well-characterised and widely leveraged carcinoma-derived tractable liver cell line. While in vitro cell lines represent a simplification of the in vivo situation, they provide a highly consistent and modifiable setting for the initial validation of type 2 diabetes variant–gene pairs in a human genetic context.

Prior work has examined the epigenomic similarities and potential causal role of type 2 diabetes-associated SNPs in EndoC-BH1 and pancreatic islets using Hi-C-based approaches [3, 6]. Complementary work with eQTLs has nominated target genes for type 2 diabetes via colocalisation and the impact of non-coding variants on transcription factor (TF) binding [5]. Moreover, EndoC-BH1 cells have been used as an in vitro platform for high-throughput perturbations of beta cell physiology, through measures of insulin secretion and content [12], providing a powerful approach for experimental validation of genome-wide predictions [8].

Building on previous reports, we describe the integration of GWAS signals for type 2 diabetes and related metabolic traits with high-resolution DpnII-based promoter-focused Capture-C in the EndoC-BH1 setting along with several other cellular models relevant to type 2 diabetes.

## Methods

### Cell culture

We used a modified protocol from the Human Cell Design (France) direct protocol for culturing EndoC-BH1 cells. Cells were expanded in DMEM low glucose (1 g/ml) (Gibco, USA) with Glutamax, 2% BSA, 50 μmol/l 2(β)-mercaptoethanol, 10 mmol/l nicotinamide, 5.5 μmol/l transferrin, 6.7 ng/ml sodium selenite, 100 U penicillin/100 μg/ml streptomycin/0.25 μg/ml amphotericin B (1×, Gibco). Plates were coated with either Matrigel or MaxGel ECM (Sigma, USA) and 2 μg/ml fibronectin according to the manufacturer’s instructions: Cells were passaged when confluence was observed or otherwise passaged by two-thirds every 2 weeks. Cells were negative for mycoplasma and validated by expression of insulin. The other cell lines were maintained under standard conditions. See the electronic supplementary material (ESM) Methods ‘Cell culture’ for details for each cell type and for the SGBS cell differentiation.

### Sequencing library preparation, sequencing and analysis

The generation of promoter-focused Capture-C, assay for transposase-accessible chromatin (ATAC-seq) and RNA-seq libraries for EndoC-BH1 was in line with methods described previously [13]. The libraries for HepG2 and a preadipocyte cell line (SGBS_undiff) that was differentiated in vitro (SGBS_diff) were generated in line with our previous reports [14]. For full details see ESM Methods ‘ATAC-seq’. We considered the set of open chromatin regions (OCRs) as the ATAC-seq peaks identified in at least two replicates in each cell line (ESM Fig. 1). We used standard methods for generation of 3C libraries [14–20]. See ESM Methods ‘Promoter-focused Capture-C’ and ‘Hi-C library preparation and analysis’ for details. The capture library was re-annotated under Gencode V30 (https://www.gencodegenes.org/) at both 1-fragment and 4-fragment resolution. We defined promoter OCRs as the set of OCRs overlapping the region within −1500/+500 bp of the gene transcription start site (TSS). This range may include both proximal and distal promoter elements but is generally not in the range where a chromatin contact at our resolution is able to distinguish these [15].

### RNA-seq library generation and analysis

RNA was isolated from ~1 million cells of each cell type using TRIzol reagent (Invitrogen, USA), purified using the Directzol RNA Miniprep Kit (Zymo Research, USA) and depleted of contaminating genomic DNA using DNAse I. Purified RNA was checked for quality on a Bioanalyzer 2100 (Agilent, USA) using the Nano RNA Chip (catalogue no. 5067-1511, Agilent) and samples with RNA integrity number (RIN) >7 were used for RNA-seq library preparation. RNA samples were depleted of ribosomal RNA using the QIAseq Fastselect RNA removal kit (Qiagen, Germany). Samples were then processed for the preparation of libraries using the SMARTer Stranded Total RNA Sample Prep Kit (Takara Bio, USA) according to the manufacturer’s instructions. See ESM Methods ‘RNA-seq’ for details.

### Bioinformatic methods

See ESM Methods ‘Partitioned LD score regression’, ‘Epigenome roadmap enrichment’, ‘GWAS data integration’ and ‘Transcription factor analysis’ for details. The resulting graphs were generated using R [21] (v4.0.2, https://www.r-project.org/) and ggplot2 (v3.3.0, Bioconductor; https://www.bioconductor.org/). Tracks were visualised using pyGenomeTracks [22] (v3.5, https://github.com/deeptools/pyGenomeTracks). Network diagrams were constructed using Cytoscape (v3.8.2, https://cytoscape.org/).

### EndoC-BH1 knockdown experiments

EndoC-βH1 cells and reagents, including the maintenance medium (OPTIβ1), starvation medium (OPTIβ2) and coating matrix (βCoat), were purchased from Human Cell Design (France). First, culture dishes were prepared following the manufacturer’s instructions and coated with βCoat. EndoC-BH1 cells were thawed in a 37°C water-bath for 1–2 min, resuspended and plated. For resuspension, 1 ml of OPTIβ1 medium was slowly added to the cells before transferring the cell suspension to a separate tube of 8 ml of OPTIβ1 medium. Following this, the cell suspension was then centrifuged for 5 min at 500 *g* at room temperature. The supernatant was discarded, and the cell pellet was resuspended with 1 ml of OPTIβ1 medium by carefully detaching the cell pellet from the bottom of the tube by gently pipetting up and down. Then, 1 ml of OPTIβ1 was added to the cell suspension and gently homogenised. Next, live cells were counted using Countess 3 (Thermo Fisher Scientific, USA). Then, 1.6 × 10^6^ cells or 4.2 × 10^6^ cells were plated in 6 cm dishes or 10 cm dishes, respectively. EndoC-βH1 cells were routinely split every 7 days to a density of 70,000 cells per cm^2^. The growth rate of EndoC-βH1 cells stabilised after the second passage of cells; therefore, all experiments were at a minimum of the third passage. The replicates were each within ±2 passages of each other.

### Glucose-stimulated insulin secretion

Prior to cell seeding, four 12-well plates were coated with 500 µl of βCoat coating matrix (Human Cell Design), following the manufacturer’s instructions. These plates were then prepared in a 37°C, 5% CO_2_ incubator for 3 h prior to cell seeding. Approximately 125,000 cells were uniformly added to each well and allowed to grow at 37°C, 5% CO_2_ for 4 days. On the fifth day the appropriate siRNA treatments (non-targeting 50 nmol/l, fragile X syndrome-related protein 2 (FXR2; 50 nmol/l) or single-pass membrane and coiled-coil domain-containing protein 4 (SMCO4; 37.5 nmol/l) were performed. For details see ESM Methods ‘EndoC-BH1 siRNA transfection’. Then, 48 h later, the cells were transferred from the siRNA/OPTIβ1 medium to OPTIβ2 starvation medium (Human Cell Design) and placed in the incubator for 24 h. Following this, the cells were preincubated for the glucose stimulation by removing the OPTIβ2 and adding 1 ml/well of βKrebs (Human Cell Design)/BSA for 60 min. Next, the stimulation step began by removing the βKrebs/BSA and adding the corresponding treatment (0 mmol/l glucose, 20 mmol/l glucose, 0 mmol/l glucose + 45 µmol/l 3-isobutyl-1-methylxanthine [IBMX] or 20 mmol/l glucose + 45 µmol/l IBMX) following manufacturer’s instructions for 40 min. For collecting the secreted insulin, 800 µl of supernatant was collected from each well and these samples were stored at 4°C until the centrifugation step. For cell insulin content, the remaining medium was removed and 1 ml of cold lysis buffer (5 mol/l NaCl, 0.2 mol/l EGTA, 1 mol/l Tris pH 8.0, glycerol, Triton X-100 and H_2_O) was added to each well for 5 min, then cells were manually sheared by pipetting up and down to complete the lysis. Next, these samples were kept at 4°C until centrifugation at 700 *g* for 5 min at 4°C. Finally, 300 µl from each sample was transferred into a new microtube and stored at −20°C until the Human Insulin ELISA was performed. For quantifying insulin content, we used the Mercodia Human Insulin ELISA kit (catalogue no. 10-1113-01, Mercodia, Sweden) to evaluate insulin secretion in EndoC-βH1 cells. Full details are available in ESM Methods ‘Human insulin ELISA’. This experiment was performed four times, with the technical replicates with the mean of each experiment treated as biological replicates.

### RNA isolation, reverse transcription and quantitative RT-PCR

Total RNA was extracted from EndoC-βH1 cells 72 h post transfection using Invitrogen TRIzol (Thermo Fisher Scientific, catalogue no. 15596018) and the Zymo Research Direct-zol RNA MiniPrep Plus kit (catalogue no. R2070), following the manufacturer’s instructions.

The quantity and purity of RNA extracted were assessed using a NanoDrop 2000 spectrophotometer (Thermo Fisher Scientific, USA).

First-strand cDNA synthesis was performed using the SuperScript VILO cDNA Synthesis Kit (Thermo Fisher Scientific, catalogue no. 11754050), following the manufacturer’s instructions. The quantity and purity of cDNA synthesised were assessed using a NanoDrop 2000 spectrophotometer.

Reverse transcriptase quantitative PCR (RT-qPCR) was performed using TaqMan Fast Advanced Master Mix (catalogue no. 4444556, Applied BioSystems, USA) and analysed with the Agilent Technologies AriaMX Real-Time System. Knockdown efficiency was assessed by RT-qPCR of the target genes using the following primers: *FXR2* (Taqman FXR2, catalogue no. 4331182, assay ID Hs00191579_m1_FAM, Thermo Fisher), *SMCO4* (Taqman SMCO4, catalogue no. 4351372, assay ID HS04980617_g1_FAM, Thermo Fisher). The endogenous control gene was beta-actin (Taqman *ACTB*, catalogue no. 4331182, assay ID HS01060665_g1_VIC, Thermo Fisher). For details see ESM Methods ‘Quantitative RT-PCR’. The endogenous control gene was used to normalise gene expression following the ΔΔC_t_ method [23]. The non-template control did not show amplification.

### Statistical analyses

Wilcoxon rank-sum tests were used to determine enrichment of expression of genes linked to promoter-interacting region OCRs (PIR-OCRs). Bar-plots with error bars represents mean and standard deviation, while bar-plots without error bars represent summary information as either a count or ratio from gene lists. In boxplots, the central line represents the median, edges represent 25th–75th percentiles, whiskers represent 1.5 times IQR and outliers are depicted as points. For experiments for glucose-stimulated insulin secretion assays, paired two-sided *t* tests were used to assess statistical significance. Randomisation was not carried out as it was not applicable to our study design and analysts were not blinded to study variables. No data were excluded from this study.

### Ethics statement

Research was conducted in accordance with the Children’s Hospital of Philadelphia’s ethics and compliance policies. The researchers had access to deidentified biospecimens and datasets.

## Results

### Epigenetic landscape of cell line models of metabolism

To gain insights into key aspects of the epigenetic landscape of type 2 diabetes-relevant cell models, we analysed high-resolution promoter-focused Capture-C and ATAC-seq data in triplicate for the EndoC-BH1 and HepG2 cell lines, along with SGBS cells both at the pre-adipocyte stage and the in vitro differentiated adipocyte-like state (SGBS_undiff, SGBS_diff). We also leveraged the gene expression in each cell line using bulk RNA-seq [14].

**Fig. 1:**
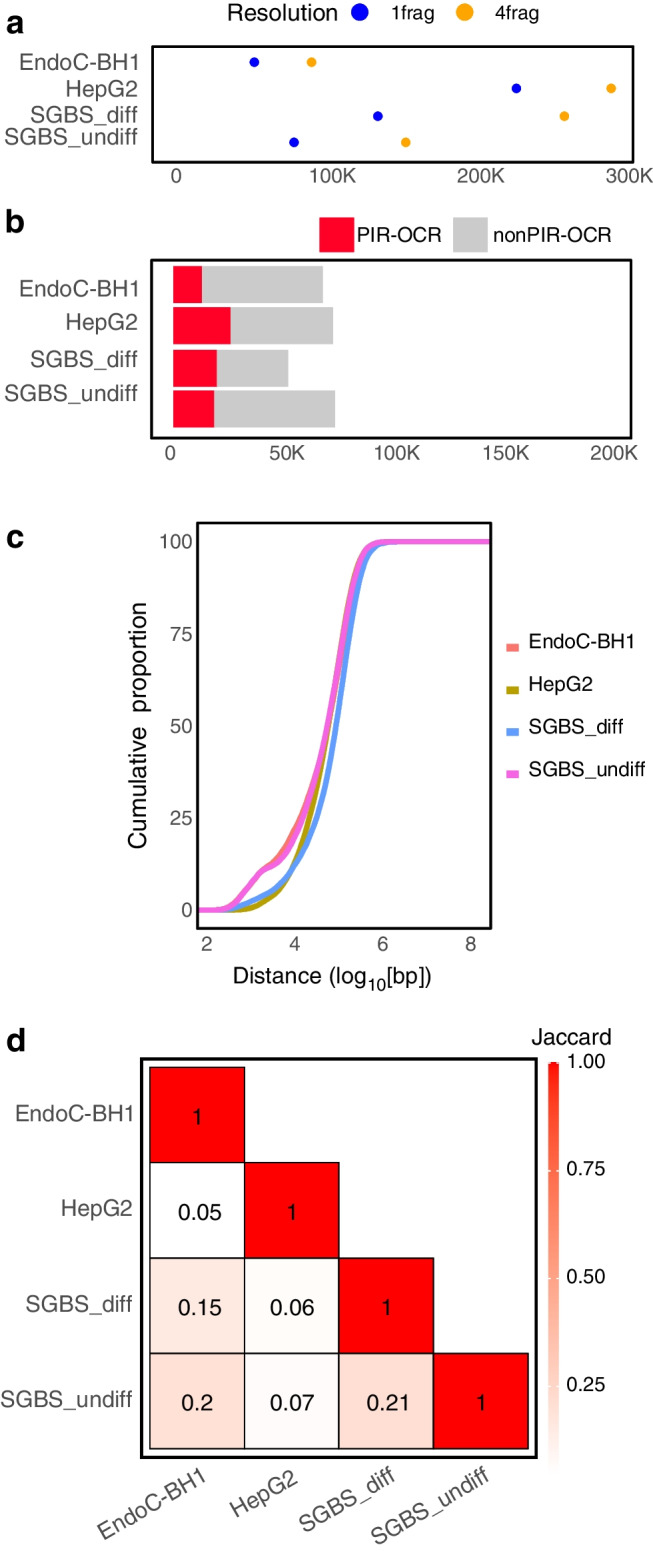
Construction of promoter interaction maps for EndoC-BH1, HepG2 and SGBS cells. (**a**) Summary of number of promoter contacts called using promoter-focused Capture-C per cell type. Fragment indicates the resolution at which the loops were called: 1frag, single fragment resolution; 4frag, binning of four consecutive fragments. (**b**) Summary of the number of OCRs called per cell type and whether the OCR intersects a PIR (PIR-OCR) or does not (nonPIR-OCR). (**c**) Cumulative distribution of distances between ends of the promoter contacts called by promoter-focused Capture-C for each cell type. (**d**) Pairwise Jaccard index of loops between promoter and OCR. Jaccard index represents the ratio of intersect to union of genomic regions in each cell type annotation

For analysing the promoter-centric chromatin architecture, we identified Capture-C-defined contacts with gene promoters both at the level of individual restriction fragments (1frag) and by in silico binning four consecutive fragments (4frag) in order to increase the power to call distant interactions, as previously performed [14, 20, 24], yielding 53,689 1frag and 92,152 4frag promoter contacts for EndoC-BH1, respectively, and ranging from 80,449 to 229,880 1frag and 155,569 to 293,618 4frag promoter contacts for the other cell lines (Fig. 1a, ESM Table 1).

We leveraged ATAC-seq to identify OCRs, which potentially act as cREs to influence gene expression. We called OCRs as the set of reproducible peaks in at least two of three replicates. We identified 68,596 OCRs in EndoC-BH1 cells, 73,296 in HepG2, 74,296 in SGBS_undiff and 52,609 in SGBS_diff.

We then annotated OCRs to genes if they either overlapped with a promoter (−1500/+500 bp) or intersected the other end called by promoter-focused Capture-C, which we termed promoter interacting regions (PIRs). We found that 28% of OCRs contacted at least one gene promoter (Fig. 1b, ESM Table 2), which is in line with previous reports [14, 20]. We next compared the distribution of distances between the putative enhancer and promoter-connected region fragments and found that they were comparable (mean: EndoC-BH1, 133,320.9 bp; HepG2, 109,703.7 bp; SGBS_undiff, 155,686.6 bp; SGBS_diff, 207,733.6 bp) (Fig. 1c).

Next, we determined the degree of similarity across promoter–OCR connections between cell types by comparing the magnitude of overlap between the PIR-OCR-connected regions using the Jaccard index, i.e. the ratio of nucleotides located in both annotations to the union present in either set of annotations. We found that the four cell types displayed less than 30% overlap (Fig. 1d), suggesting that the promoter landscapes have distinct cell type-specific epigenetic and chromatin conformational features that contribute to transcriptional differences across these cell lines. Also consistent with previous reports, we observed bait-to-bait contacts ranging from 10% to 29% of called loops (ESM Table 3).

### Comparison of promoter-focused defined maps with existing enhancer atlases

**Fig. 2:**
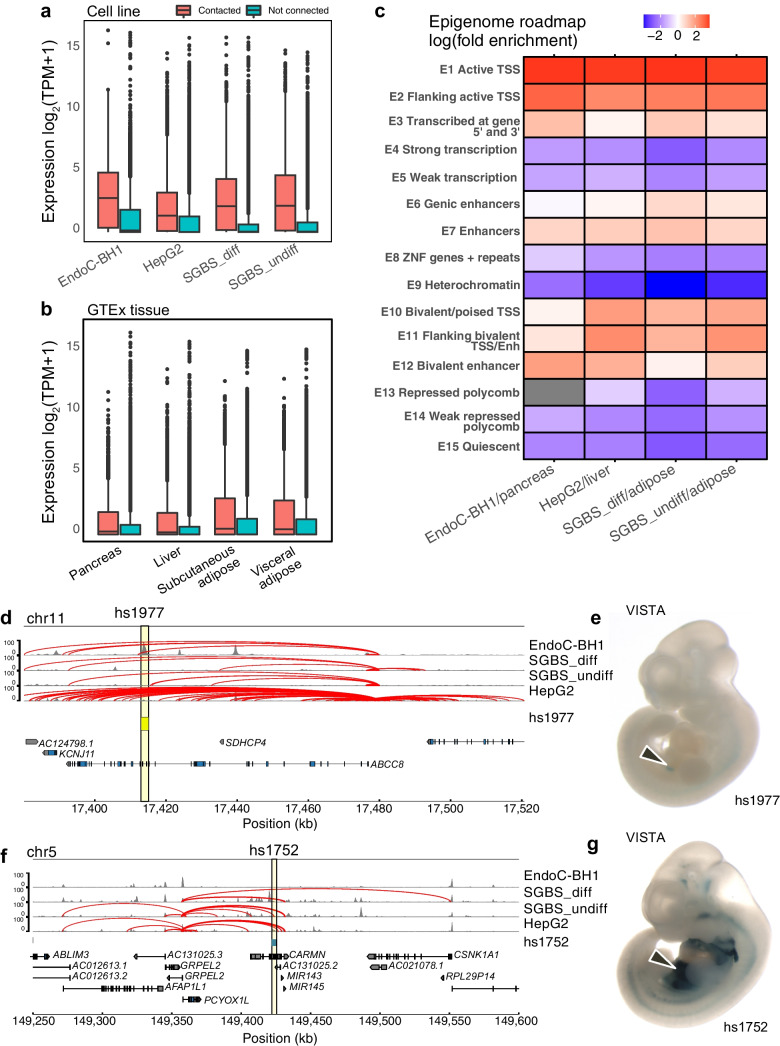
Validation of promoter-connected cREs. (**a**) The distribution of gene expression from matching cell line data (log_2_ TPM+1) between genes with promoters with at least one PIR-OCR contact (red) and those genes without PIR-OCRs (green). (**b**) The distribution of gene expression from GTEx tissue (log_2_ TPM+1) from the cells between genes with promoters with at least one PIR-OCR contact (red) and those genes without PIR-OCRs (green). Pancreas expression with EndoC-BH1 contacts; visceral/subcutaneous adipose tissue expression with SGBS_diff and SGBS_undiff contacts; and liver expression with HepG2 contacts. In boxplots, the central line represents the median, box edges represent 25th–75th percentiles, whiskers represent 1.5 times the IQR and outliers are depicted as points. (**c**) log_2_-fold enrichment of PIR-OCRs to ChromHMM-defined annotations for the epigenome roadmap for the indicated cell type/tissue type pairs. (**d**) Genomic location of a verified pancreas enhancer in beta cells from the VISTA regulatory element browser with chromatin contacts to *ABCC8.* (**e**) *lacZ* staining from the VISTA browser showing the expression pattern for the contact between *ABCC8* and hs1977 detailed in (**d**). (**f**) Genomic location of a liver-expressed regulatory element from VISTA with chromatin contacts to *PCYOX1L* in HepG2 cells (**g**) *lacZ* staining from the VISTA browser showing the expression pattern for the contact between *PCYOX1L* and hs1752

To further validate our OCR–gene contacts, we compared the expression of genes with a promoter contacting at least one OCR vs that of genes not in contact with any OCR. Consistent with our prior promoter-focused Capture-C datasets [16, 17, 19], genes with a promoter in contact with OCRs were generally expressed at significantly higher levels (unpaired Mann–Whitney *U* test, two-sided *p*<2.2 × 10^−16^) than those without OCR contacts across cell types (Fig. 2a). Similarly, we assessed the degree of similarity to GTEx expression profiles of equivalent tissue types and found that the genes contacted in our cell lines were expressed at higher levels in the corresponding tissue in that public domain dataset [25] (Fig. 2b). These results suggest that promoter contacts with OCRs are associated with increased gene expression.

We then compared the chromatin states identified by the epigenome roadmap with the open connected regions defined by ATAC-seq and promoter-focused Capture-C. As expected, we found at least fourfold enrichment for active and bivalent TSSs and enhancers, given that OCRs can represent either active or ‘poised’ regulatory elements (Fig. 2c). The shared TSS/promoter signature represents the number of bait-to-bait interactions present, suggesting either that some genes are co-regulated or that promoters of genes not expressed in a particular cell type can act as enhancers, as previously reported by others [26].

We subsequently examined the VISTA database for curated validated enhancers in different embryological tissues [27]. However, there were only six experimentally validated pancreatic enhancers within the database. Despite this limitation, we observed in our data that a known human enhancer, hs1977, was in contact with the *ABCC8* promoter (Fig. 2d, e). *ABCC8* encodes a component of an ATP-sensitive potassium channel expressed in beta cells and modulates glucose-dependent insulin secretion [28]. This enhancer region was accessible only in EndoC-BH1 cells, while expression was observed in the pancreas in the VISTA database, although several of the in situs display staining in neural tissues in addition to the pancreas. We also identified a liver enhancer, hs1752, expressed in the liver, heart and other abdominal tissues with contacts to *PCYOX1L*, which encodes prenylcysteine oxidase 1 like (Fig. 2f, g). Prenylcysteine oxidases are enzymes that scavenge free cysteines from a metabolic pathway involved in the degradation of prenylated proteins [29]. This cRE was also connected to the promoter of several non-coding RNAs: miR-143, miR-145 and AC131025.2. Taken together, these results support the use of chromatin features in these cell lines as a valid model to investigate cREs for type 2 diabetes-related traits.

### Variant-to-gene mapping in metabolic-relevant cells and prioritisation of type 2 diabetes-associated GWAS signals

**Fig. 3:**
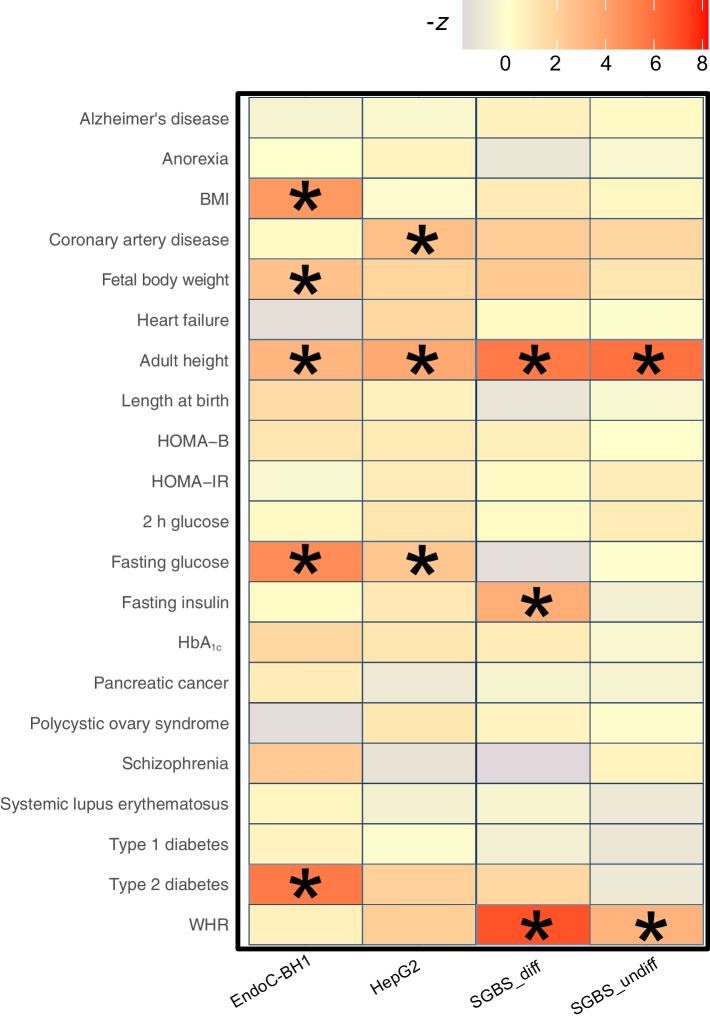
Enrichment of metabolic traits in EndoC-BH1 and relevant cells. A summary of partitioned LD score regression showing the *z* score of enrichment for indicated traits. Colour indicates the *z*-transformed *p* values, with traits significantly enriched marked with asterisks

Next, we sought to investigate the degree that cellular models are enriched for heritability associated with metabolic traits. To this end, we performed partitioned LD score regression [30] to test for enrichment of putative cREs (promoter OCRs + PIR-OCRs) (Fig. 3, ESM Table 4). We observed significant enrichment of EndoC-BH1 cREs for type 2 diabetes, fasting glucose, BMI and fetal body weight. HepG2 cREs were enriched for coronary artery disease and fasting glucose levels. SGBS_diff cREs were enriched for WHR, fasting insulin, plasma triglyceride and HDL-cholesterol levels, while SGBS_undiff cREs were enriched for WHR. cREs across all cell types were enriched for height. In addition, we included recent GWAS studies from Alzheimer’s disease and systemic lupus erythematosus as negative controls, and as expected we did not observe enrichment for these neural and immune GWAS traits in these principally metabolic cell types [30–32]. Therefore, these results supported the utility of these cell models in investigating the *cis*-regulatory architecture for the type 2 diabetes-related traits.

**Fig. 4:**
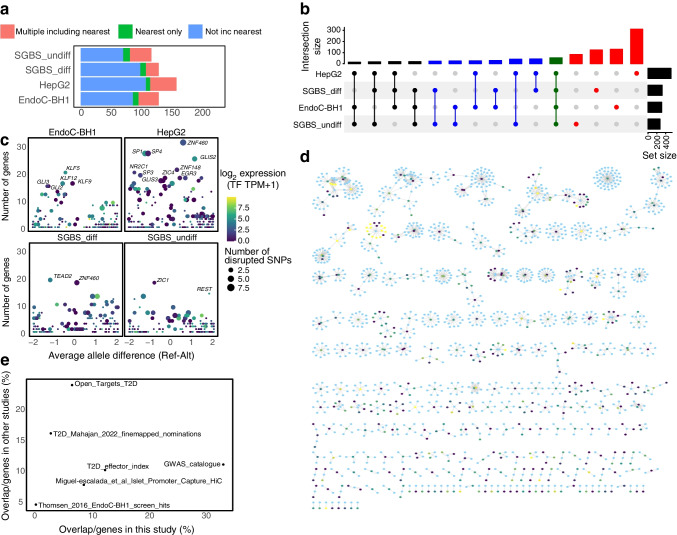
V2G mapping of type 2 diabetes loci across cell types. (**a**) The counts of sentinels implicated to the nearest gene to the sentinel, to multiple genes including the nearest gene or to gene(s) not including the gene closest to sentinel for each cell type. (**b**) Overlap of genes implicated in type 2 diabetes in each cell type; the top bar graph indicates the number of genes in each intersection set (intersection size). Red indicates the subset of genes found in one cell type, blue indicates two cell types, black indicates three cell types and green indicates the genes implicated in all four cell types. The side bar graph indicates the total number of genes per cell type (set size). (**c**) TFs predicted to be impacted by proxy SNPs. The *x*-axis indicates whether the mean effect of type 2 diabetes SNPs is predicted to be increasing or decreasing stability and the *y*-axis indicates the expression of the TF. Colour is scaled to indicate the mean expression of the predicted target genes and size indicates the number of proxies predicted to disrupt a given TF motif. (**d**) Network showing predicted proxies (blue) connected by promoter-focused Capture-C and ATAC-seq to target genes (coloured by expression: blue, low; yellow, higher expression). (**e**) Intersection of our list of implicated genes in EndoC-BH1s with several known databases. T2D_Mahajan_2022_finemapped_nominations [36]; Open_Targets_T2D [46]; T2D_effector_index [37]; GWAS_catalogue [47]; Miguel-Escalada_et_al_Islet_Promoter_Capture_HiC [3]; Thomsen_2016_EndoC-BH1_screen_hits [8]. Ref-Alt, reference allele minus alternative allele; T2D, type 2 diabetes

To implicate type 2 diabetes causal variants impacting cREs, we curated the set of lead sentinel SNPs from the two most recent European and trans-ancestral GWAS reports for the disease [33, 34]. We identified proxies in high LD with the reported lead variants for each signal (*r*^2^>0.8) and intersected their genomic coordinates with OCRs and PIRs (Table 1). The subsequent 810 implicated genes from this variant-to-gene (V2G) mapping strategy, corresponding to 370 type 2 diabetes sentinels, revealed that the majority of putative causal variants did not contact the gene nearest to the sentinel, and in most cases where the nearest gene was implicated other gene promoters were also additionally contacted (Fig. 4a, ESM Table 5). Of these 810 genes implicated by this V2G mapping approach, 79.9% were specific to one cell line (130 EndoC-BH1, 311 HepG2, 123 SGBS_diff, 83 SGBS_undiff) while 163 (20.1%) were shared in at least two cell types and 53 (6.5%) across all cell types (Fig. 4b). We compared the list of genes with phenotypes identified in mice, and found significant enrichment for nervous system, haematopoietic system, growth/size body region and mortality/ageing (ESM Fig. 2, ESM Table 6).

**Table 1:** Summary table of the input sentinels and proxies used in this study and how many in at least one cell type

Variable	Mahajan et al, 2018 [36]	Vujkovic et al, 2020 [45]				
Ancestry	EUR	AFR	AMR	EAS	EUR	TRANSE
No. of sentinel SNPs	403	21	2	86	425	553
No. of proxy SNPs	8781	129	20	2253	10,339	10,893
No. of PIR-OCR proxy SNPs	314	2	5	72	301	271

AFR, African ancestry analysis; AMR, American ancestry analysis; EAS, East Asian ancestry analysis; EUR, European ancestry analysis; TRANSE, trans-ancestral analysis

One obvious mechanism by which non-coding variants can influence gene expression is through disruption of TF binding sites. To investigate this possibility, we predicted whether any of the V2G-implicated open proxies that contacted genes contained known TF binding motifs. This approach led to the identification of 288 proxies predicted to alter binding affinity (Fig. 4c, ESM Table 7). Most notably, we observed that several binding sites for zinc finger TF KLF and GLI families were disrupted by variants identified in EndoC-BH1. Seventeen of the TFs with motifs impacted by type 2 diabetes variants are predicted effector transcripts in at least one cell type (*HES2*, *ZNF384*, *STAT6*, *NR2C1*, *ONECUT1*, *TCF12*, *SOX15*, *TP53*, *HNF1B*, *TCF4*, *TCF3*, *OSR1*, *PPARG*, *NFKB1*, *CREB3*, *ZBTB6*, *ZBTB26*). We compared the motifs with publicly available chromatin immunoprecipitation (ChIP) data from ENCODE, to determine if there is evidence of TF binding at the genomic location in various cellular contexts (ESM Fig. 3, ESM Table 8). We also checked the expression of implicated TFs and observed 280 of 447 (62.6%) of the predicted TFs with disrupted motifs had transcripts per million mapped reads (TPM) >1 and 187 had TPM >10 in EndoC-BH1 cells, suggesting that a majority of predicted TFs are expressed (ESM Fig. 4).

We then examined the genes contacting proxies regardless of the status of predicted disruption of TF binding sites. While some signals were only predicted to have one proxy interacting with one gene, others yielded multiple proxies with contacts to multiple genes (Fig. 4d). While this could suggest a subset of variants acting through multiple genes, further work is necessary to functionally validate these predictions. We note that we did not observe a trend towards those multi-gene signals corresponding to more statistically significant type 2 diabetes GWAS loci.

Next, we compared our analysis with previous type 2 diabetes functional prioritisation approaches, yielding moderate agreement but highlighting a potentially higher confidence gene set for further investigation [3, 8, 35–37] (Fig. 4e, ESM Table 9). Additionally, we compared our findings with a recent CRISPR interference (CRISPRi) screen identifying potential type 2 diabetes effectors, noting eight overlapping genes, seven associated with decreased insulin content and one with increased content (decreased: *CHD4*, *PRPF18*, *GMEB1*, *CREB3*, *PITPNM2*, *SIN3A* and *ATP6 V1C1*; increased: *FADS1*). However, this overlap did not reach strict statistical significance (Fisher’s test, one-sided, *p*=0.09). Moreover, we compared our list with one generated using chromatin conformation in pancreatic cells, finding 100 genes annotated as type 2 diabetes effectors in beta cell Hi-C also present in our dataset (ESM Table 10) [9].

Subsequently, we compared our results with eQTL associations of nearby genes [5, 25]. Among 221 eQTL-linked genes associated with type 2 diabetes risk loci in pancreatic/islet tissues, only 12 had open proxies in chromatin contact with their promoters in EndoC-BH1 cells: *SMCO4*, *DOC2A*, *OPRL1*, *TUFM*, *YBEY*, *MTMR11*, *UBE2D3*, *PLEKHA1*, *GPSM1*, *RNF6*, *ABCD9* and *AGFG2*. Six genes were implicated by both liver eQTLs and our promoter-focused Capture-C in HepG2 cells, while 25 genes were linked to adipose tissue eQTLs and our Capture-C in at least one adipose model (SGBS_undiff, SGBS_diff) (ESM Table 11). Colocalisation analyses between GTEx v7 and DIAMANTE type 2 diabetes GWAS signals [33] revealed 22 genes colocalised (posterior inclusion probability (PIP) >0.85) in the pancreas, with only *DOC2A* also implicated by our Capture-C approach. We detected 11 genes with eQTL colocalisation in the liver, of which *MAN2C1*, *AP3S2* and *CEP68* were also implicated by promoter contacts in HepG2. Additionally, 54 genes were identified in subcutaneous or visceral adipose tissue, with *AP3S2*, *CALR* and *NDUFAF6* also implicated by our V2G approach in SGBS_diff cells, while *PABPC4*, *PLEKHA1*, *AP3S2* and *DCAF16* were implicated in the SGBS_undiff dataset (ESM Table 12). Although there is limited overlap, the intersection of these methods can better prioritise genes for functional validation.

### Inhibition of* SMCO4* expression increases glucose-stimulated insulin secretion in EndoC-BH1

**Fig. 5:**
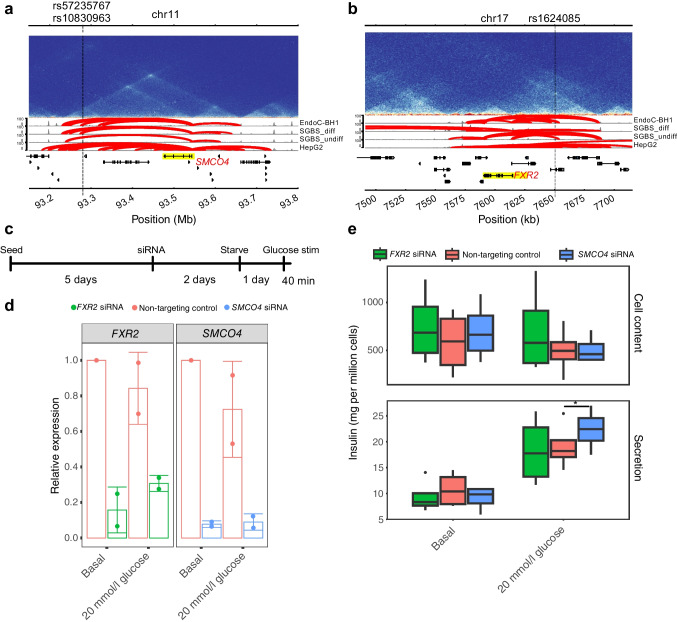
Assaying effects of *SMCO4* and *FXR2* knockdown in Endo-BH1 cells on glucose-stimulated insulin secretion. (**a**, **b**) Genomic locations of *SMCO4* (**a**) and *FXR2* (**b**) relative to implicated proxy variants. The top tracks represent Hi-C contact matrices from EndoC-BH1 Hi-C data, while the subsequent tracks depict significant contacts for Capture-C (Chicago score ≥5) and ATAC-seq (normalised using the reads-per-genomic-content approach). The locations of implicated SNPs are drawn as vertical lines. (**c**) Diagram summarising the time course of insulin treatment. (**d**) RT-qPCR results of the knockdown of either *FXR2* or *SMCO4*. The data were adjusted using the ΔΔC_t_ method and all conditions were subsequently normalised to basal non-targeting control. Two datapoints are depicted and the top of the bar graph depicts the mean expression and error bars depict the SD. (**e**) Boxplots depict the results of the insulin secretion assay, the central line represents the median, box edges represent 25th–75th percentiles, whiskers represent 1.5 times the IQR and outliers are depicted as points. Insulin content and secretion were calculated from ELISA plates with standard curves (see Methods). Measurements were taken for either basal (0 mmol/l glucose) or 20 mmol/l glucose. The mean of six technical replicates was taken for four biological replicates. Paired two-tailed *t* tests were used to assess statistical significance across the four biological replicates. *p<0.05. Stim, stimulation

In our analysis pipeline, two genes, *SMCO4* and *FXR2*, were among those identified as potential type 2 diabetes candidate genes (Fig. 5a, b) in both EndoC-BH1 and prior primary beta cell chromatin maps [9]. Additionally, an eQTL associated with *SMCO4* colocalises with a type 2 diabetes signal marked by rs57235767 in islets [5]. To investigate the impact of these two genes on insulin secretion in EndoC-BH1 cells (Fig. 5c), we performed targeted knockdown experiments of *SMCO4* and *FXR2*. Confirmation of *SMCO4* and *FXR2* knockdown was achieved using RT-qPCR (Fig. 5d). Our analyses revealed a significant increase in insulin secretion in *SMCO4* knockdown cells relative to non-targeting siRNA controls (~18.0% increase, paired two-tailed *t* test *p*=0.0243) (Fig. 5e). Notably, there was no significant difference when IBMX was added to the stimulation medium (ESM Fig. 5). IBMX raises cellular levels of cAMP to stimulate secretion of insulin. These results suggest that that *SMCO4*, but not *FXR2*, inhibits glucose-stimulated insulin secretion in EndoC-BH1 cells, while having no significant impact on IBMX-stimulated secretion.

## Discussion

Through the integration of GWAS data with the data generation of the trifecta of promoter-focused Capture-C, ATAC-seq and RNA-seq in type 2 diabetes-related relevant cellular models, we could reveal which settings showed enrichment for loci and V2G genes for subsequent informing of functional studies to pursue candidate causal variants and their corresponding effector genes. Given that prior studies have examined putative effectors of type 2 diabetes-associated SNPs in pancreatic islets using eQTLs and promoter Hi-C [3, 5, 6], along with similar work in adipose [38] and skeletal muscle cells [39], our work complements previous efforts with cellular models particularly amenable for functional experiments to address such complex, polygenic diseases operating across multiple tissue settings.

Partitioned LD score regression analyses revealed differences in cellular composition for metabolic-related traits. Type 2 diabetes and fasting glucose displayed enrichment in EndoC-BH1 cells, which supports evidence from prior studies highlighting the pancreas's role in glucose regulation [7]. BMI enrichment in EndoC-BH1 cells is consistent with the insulin–obesity association [40]. Height exhibited enrichment across all cell types, possibly due to the large sample size and its reflection of various physiological processes [41]. Fasting insulin levels and WHR were enriched in the adipose model SGBS_diff, indicating differing genetic impacts on metabolic physiology between type 2 diabetes and associated risk factors (Fig. 3). No enrichment was found for polycystic ovary syndrome, a disorder leading to infertility that often presents with insulin resistance [42].

V2G mapping identified *SMCO4* as a candidate gene affecting insulin secretion. From public expression resources, *SMCO4* is expressed in most tissues from RNA-seq and immunohistochemistry data [43]. Further work would be necessary to characterise its role in pancreatic islet cells and other tissues. In addition, we queried several prior studies and found that SMCO4 is not reported to be differentially expressed in islets/beta cells in type 2 diabetes. It is possible that SMCO4 functions early in type 2 diabetes development or in a limited subset of cells. *FXR2* knockdown showed less efficiency and no significant effect on insulin secretion, possibly due to either reduced knockdown efficiency or compensation by its paralog *FXR1*.

Despite the valuable insights gained, our study has notable limitations. While commonly used for molecular validation of disease variants, the use of immortalised cell lines does not fully recapitulate the chromatin landscape of the in vivo state of primary cells/tissues. Moreover, these cells provide snapshots of cellular states under limited conditions and do not take into account possible temporal effects at loci over time. Incorporating multiple lines of evidence facilitates determination of causality of disease-relevant variants. Further comparative analyses under different physiological conditions may offer additional insights into gene expression programmes and expand the list of nominated genes. In our functional assays, while we successfully validated *SMCO4* knockdown, we faced challenges in assaying SMCO4 protein knockdown via western blot due to the small size and negative charge of the protein. Further work is warranted to further clarify the function of SMCO4 in beta cells in the context of type 2 diabetes and related traits. In addition, we only assayed for functional consequences of putative effector genes in EndoC-BH1 cells. Future work could assay responsiveness to insulin via downstream molecular readouts and other physiological responses in SGBS and HepG2 cells.

The combined results from our V2G mapping analyses present potential targets for further validation and functional assessment in cellular models, especially when combined with other orthogonal datasets available in the public domain. Additional work is necessary to determine whether these observations represent true cases of pleiotropy. Our datasets were generated to capture the ‘normal context’ of disease-associated susceptibility variants before the disease induces thousands of non-causal changes after onset. However, it has been proposed that some SNPs may act by retethering regulatory elements to ectopic target genes to contribute to disease [44]. High-throughput functional validation methods, such as Perturb-seq, may offer insights into how eQTLs, chromatin conformation and other data contribute to effector gene nominations. Future studies focused on highlighting the dynamic nature of enhancers in a variety of cellular states and disease contexts are warranted, as well as comparing the 3D architecture of larger cohorts with diverse genetic backgrounds, in order to clarify how generalised this model is in shedding light on the genomic aetiology of common metabolic disease pathogenesis.

## Supplementary Information

Below is the link to the electronic supplementary material.

ESM (PDF 478 KB)

ESM Tables (XLSX 21628 KB)

## Data Availability

Raw and processed next-generation sequencing data for EndoC-BH1, HepG2, SGBS_undiff and SGBS_diff cells are deposited in GEO under the Superseries accession GSE262484. Promoter-focused Capture-C data are deposited under accession GSE262496. Hi-C data are deposited under accession GSE262481. Bulk ATAC-seq data are deposited under accession GSE262479. Bulk RNA-seq data are deposited under accession GSE262480.

## Abbreviations

ATAC-seq: Assay for transposase-accessible chromatin with sequencing
cRE: *Cis*-regulatory element
eQTL: Expression quantitative trait locus
1frag: One fragment; individual DpnII restriction fragment
4frag: Four fragments; four sequential DpnII restriction fragments concatenated in silico
FXR2: Fragile X syndrome-related protein 2
GWAS: Genome-wide association study
IBMX: 3-Isobutyl-1-methylxanthine
LD: Linkage disequilibrium
OCR: Open chromatin region
PIR: Promoter interacting region
PIR-OCR: Promoter-interacting region OCR
RNAi: RNA interference
RIN: RNA Integrity Number
RT-qPCR: Reverse transcriptase quantitative PCR
SGBS: Simpson–Golabi–Behmel syndrome preadipocyte cell line
SGBS_diff: Differentiated SGBS cells
SGBS_undiff: Undifferentiated SGBS cells
SMCO4: Single-pass membrane and coiled-coil domain-containing protein 4
TF: Transcription factor
TPM: Transcripts per million mapped reads
TSS: Transcription start site
V2G: Variant-to-gene

## References

[1] Jerkovic I, Cavalli G (2021) Understanding 3D genome organization by multidisciplinary methods. Nat Rev Mol Cell Biol 22(8):511–528. 10.1038/s41580-021-00362-w 33953379 10.1038/s41580-021-00362-w

[2] Schoenfelder S, Fraser P (2019) Long-range enhancer-promoter contacts in gene expression control. Nat Rev Genet 20(8):437–455. 10.1038/s41576-019-0128-0 31086298 10.1038/s41576-019-0128-0

[3] Miguel-Escalada I, Bonas-Guarch S, Cebola I et al (2019) Human pancreatic islet three-dimensional chromatin architecture provides insights into the genetics of type 2 diabetes. Nat Genet 51(7):1137–1148. 10.1038/s41588-019-0457-0 31253982 10.1038/s41588-019-0457-0 PMC6640048

[4] Javierre BM, Burren OS, Wilder SP et al (2016) Lineage-specific genome architecture links enhancers and non-coding disease variants to target gene promoters. Cell 167(5):1369-1384 e1319. 10.1016/j.cell.2016.09.037 27863249 10.1016/j.cell.2016.09.037 PMC5123897

[5] Varshney A, Scott LJ, Welch RP et al (2017) Genetic regulatory signatures underlying islet gene expression and type 2 diabetes. Proc Natl Acad Sci U S A 114(9):2301–2306. 10.1073/pnas.1621192114 28193859 10.1073/pnas.1621192114 PMC5338551

[6] Lawlor N, Marquez EJ, Orchard P et al (2019) Multiomic profiling identifies cis-regulatory networks underlying human pancreatic β cell identity and function. Cell Rep 26(3):788-801 e786. 10.1016/j.celrep.2018.12.083 30650367 10.1016/j.celrep.2018.12.083 PMC6389269

[7] Chiou J, Zeng C, Cheng Z et al (2021) Single-cell chromatin accessibility identifies pancreatic islet cell type- and state-specific regulatory programs of diabetes risk. Nat Genet 53(4):455–466. 10.1038/s41588-021-00823-0 33795864 10.1038/s41588-021-00823-0 PMC9037575

[8] Thomsen SK, Ceroni A, van de Bunt M et al (2016) Systematic functional characterization of candidate causal genes for type 2 diabetes risk variants. Diabetes 65(12):3805–3811. 10.2337/db16-0361 27554474 10.2337/db16-0361 PMC5402869

[9] Su C, Gao L, May CL et al (2022) 3D chromatin maps of the human pancreas reveal lineage-specific regulatory architecture of T2D risk. Cell Metab 34(9):1394-1409 e1394. 10.1016/j.cmet.2022.08.014 36070683 10.1016/j.cmet.2022.08.014 PMC9664375

[10] Tsonkova VG, Sand FW, Wolf XA et al (2018) The EndoC-βH1 cell line is a valid model of human beta cells and applicable for screenings to identify novel drug target candidates. Mol Metab 8:144–157. 10.1016/j.molmet.2017.12.007 29307512 10.1016/j.molmet.2017.12.007 PMC5985049

[11] Wabitsch M, Melzner I, Braun M et al (2001) Characterization of a human preadipocyte cell strain with high capacity for adipose differentiation. Int J Obes 25:8–15. 10.1038/sj.ijo.0801520 10.1038/sj.ijo.0801520 11244452

[12] Rottner AK, Ye Y, Navarro-Guerrero E et al (2023) A genome-wide CRISPR screen identifies CALCOCO2 as a regulator of beta cell function influencing type 2 diabetes risk. Nat Genet 55(1):54–65. 10.1038/s41588-022-01261-2 36543916 10.1038/s41588-022-01261-2 PMC9839450

[13] Lasconi C, Pahl MC, Pippin JA et al (2022) Variant-to-gene-mapping analyses reveal a role for pancreatic islet cells in conferring genetic susceptibility to sleep-related traits. Sleep 45(8):zsac109. 10.1093/sleep/zsac109 35537191 10.1093/sleep/zsac109 PMC9366645

[14] Chesi A, Wagley Y, Johnson ME et al (2019) Genome-scale Capture C promoter interactions implicate effector genes at GWAS loci for bone mineral density. Nat Commun 10(1):1260. 10.1038/s41467-019-09302-x 30890710 10.1038/s41467-019-09302-x PMC6425012

[15] Caliskan M, Manduchi E, Rao HS et al (2019) Genetic and epigenetic fine mapping of complex trait associated loci in the human liver. Am J Hum Genet 105(1):89–107. 10.1016/j.ajhg.2019.05.010 31204013 10.1016/j.ajhg.2019.05.010 PMC6612522

[16] Pahl MC, Doege CA, Hodge KM et al (2021) Cis-regulatory architecture of human ESC-derived hypothalamic neuron differentiation aids in variant-to-gene mapping of relevant complex traits. Nat Commun 12(1):6749. 10.1038/s41467-021-27001-4 34799566 10.1038/s41467-021-27001-4 PMC8604959

[17] Pahl MC, Le Coz C, Su C et al (2022) Implicating effector genes at COVID-19 GWAS loci using promoter-focused Capture-C in disease-relevant immune cell types. Genome Biol 23(1):125. 10.1186/s13059-022-02691-1 35659055 10.1186/s13059-022-02691-1 PMC9164584

[18] Palmero J, Chesi A, Zimmerman A et al (2023) Variant-to-gene mapping followed by cross-species genetic screening identifies GPI-anchor biosynthesis as a regulator of sleep. Sci Adv 9:eabq0844. 10.1126/sciadv.abq0844 36608130 10.1126/sciadv.abq0844 PMC9821868

[19] Su C, Argenziano M, Lu S et al (2021) 3D promoter architecture re-organization during iPSC-derived neuronal cell differentiation implicates target genes for neurodevelopmental disorders. Prog Neurobiol 201:102000. 10.1016/j.pneurobio.2021.102000 33545232 10.1016/j.pneurobio.2021.102000 PMC8096691

[20] Su C, Johnson ME, Torres A et al (2020) Mapping effector genes at lupus GWAS loci using promoter Capture-C in follicular helper T cells. Nat Commun 11(1):3294. 10.1038/s41467-020-17089-5 32620744 10.1038/s41467-020-17089-5 PMC7335045

[21] R Core Team (2021) R: A language and environment for statistical computing. R Foundation for Statistical Computing, Vienna, Austria

[22] Lopez-Delisle L, Rabbani L, Wolff J et al (2021) pyGenomeTracks: reproducible plots for multivariate genomic datasets. Bioinformatics 37(3):422–423. 10.1093/bioinformatics/btaa692 32745185 10.1093/bioinformatics/btaa692 PMC8058774

[23] Schmittgen TD, Livak KJ (2008) Analyzing real-time PCR data by the comparative C_T_ method. Nat Protoc 3(6):1101–1108. 10.1038/nprot.2008.73 18546601 10.1038/nprot.2008.73

[24] Su C, Pahl MC, Grant SFA, Wells AD (2021) Restriction enzyme selection dictates detection range sensitivity in chromatin conformation capture-based variant-to-gene mapping approaches. Hum Genet 140(10):1441–1448. 10.1007/s00439-021-02326-8 34405268 10.1007/s00439-021-02326-8 PMC9013487

[25] GTeX Consortium (2017) Genetic effects on gene expression across human tissues. Nature 550(7675):204–213. 10.1038/nature24277 29022597 10.1038/nature24277 PMC5776756

[26] Jung I, Schmitt A, Diao Y et al (2019) A compendium of promoter-centered long-range chromatin interactions in the human genome. Nat Genet 51(10):1442–1449. 10.1038/s41588-019-0494-8 31501517 10.1038/s41588-019-0494-8 PMC6778519

[27] Visel A, Minovitsky S, Dubchak I, Pennacchio LA (2007) VISTA Enhancer Browser a database of tissue-specific human enhancers. Nucleic Acids Res 35(Database):D88–D92. 10.1093/nar/gkl822 17130149 10.1093/nar/gkl822 PMC1716724

[28] Ahlqvist E, Ahluwalia TS, Groop L (2011) Genetics of type 2 diabetes. Clin Chem 57(2):241–254. 10.1373/clinchem.2010.157016 21119033 10.1373/clinchem.2010.157016

[29] Tschantz WR, Zhang L, Casey PJ (1999) Cloning, expression, and cellular localization of a human prenylcysteine lyase. J Biol Chem 274(50):35802–35808. 10.1074/jbc.274.50.35802 10585463 10.1074/jbc.274.50.35802

[30] Finucane HK, Bulik-Sullivan B, Gusev A et al (2015) Partitioning heritability by functional annotation using genome-wide association summary statistics. Nat Genet 47(11):1228–1235. 10.1038/ng.3404 26414678 10.1038/ng.3404 PMC4626285

[31] Timshel PN, Thompson JJ, Pers TH (2020) Genetic mapping of etiologic brain cell types for obesity. Elife 9:e55851. 10.7554/eLife.55851 32955435 10.7554/eLife.55851 PMC7505664

[32] Locke AE, Kahali B, Berndt SI et al (2015) Genetic studies of body mass index yield new insights for obesity biology. Nature 518(7538):197–206. 10.1038/nature14177 25673413 10.1038/nature14177 PMC4382211

[33] Mahajan A, Taliun D, Thurner M et al (2018) Fine-mapping type 2 diabetes loci to single-variant resolution using high-density imputation and islet-specific epigenome maps. Nat Genet 50(11):1505–1513. 10.1038/s41588-018-0241-6 30297969 10.1038/s41588-018-0241-6 PMC6287706

[34] Vujkovic M, Keaton JM, Lynch JA et al (2020) Discovery of 318 new risk loci for type 2 diabetes and related vascular outcomes among 1.4 million participants in a multi-ancestry meta-analysis. Nat Genet 52(7):680–691. 10.1038/s41588-020-0637-y 32541925 10.1038/s41588-020-0637-y PMC7343592

[35] Mountjoy E, Schmidt EM, Carmona M et al (2021) An open approach to systematically prioritize causal variants and genes at all published human GWAS trait-associated loci. Nat Genet 53(11):1527–1533. 10.1038/s41588-021-00945-5 34711957 10.1038/s41588-021-00945-5 PMC7611956

[36] Mahajan A, Spracklen CN, Zhang W et al (2022) Multi-ancestry genetic study of type 2 diabetes highlights the power of diverse populations for discovery and translation. Nat Genet 54(5):560–572. 10.1038/s41588-022-01058-3 35551307 10.1038/s41588-022-01058-3 PMC9179018

[37] Forgetta V, Jiang L, Vulpescu NA et al (2022) An effector index to predict target genes at GWAS loci. Hum Genet 141(8):1431–1447. 10.1007/s00439-022-02434-z 35147782 10.1007/s00439-022-02434-z

[38] Pan DZ, Garske KM, Alvarez M et al (2018) Integration of human adipocyte chromosomal interactions with adipose gene expression prioritizes obesity-related genes from GWAS. Nat Commun 9(1):1512. 10.1038/s41467-018-03554-9 29666371 10.1038/s41467-018-03554-9 PMC5904163

[39] Williams K, Ingerslev LR, Bork-Jensen J et al (2020) Skeletal muscle enhancer interactions identify genes controlling whole-body metabolism. Nat Commun 11(1):2695. 10.1038/s41467-020-16537-6 32483258 10.1038/s41467-020-16537-6 PMC7264154

[40] Astley CM, Todd JN, Salem RM et al (2018) Genetic evidence that carbohydrate-stimulated insulin secretion leads to obesity. Clin Chem 64(1):192–200. 10.1373/clinchem.2017.280727 29295838 10.1373/clinchem.2017.280727 PMC5937525

[41] Yengo L, Sidorenko J, Kemper KE et al (2018) Meta-analysis of genome-wide association studies for height and body mass index in approximately 700000 individuals of European ancestry. Hum Mol Genet 27(20):3641–3649. 10.1093/hmg/ddy271 30124842 10.1093/hmg/ddy271 PMC6488973

[42] Day F, Karaderi T, Jones MR et al (2018) Large-scale genome-wide meta-analysis of polycystic ovary syndrome suggests shared genetic architecture for different diagnosis criteria. PLoS Genet 14(12):e1007813. 10.1371/journal.pgen.1007813 30566500 10.1371/journal.pgen.1007813 PMC6300389

[43] Thul PJ, Lindskog C (2018) The human protein atlas: a spatial map of the human proteome. Protein Sci 27(1):233–244. 10.1002/pro.3307 28940711 10.1002/pro.3307 PMC5734309

[44] Oh S, Shao J, Mitra J et al (2021) Enhancer release and retargeting activates disease-susceptibility genes. Nature 595(7869):735–740. 10.1038/s41586-021-03577-1 34040254 10.1038/s41586-021-03577-1 PMC11171441

[45] Vujkovic M, Ramdas S, Lorenz KM et al (2022) A multiancestry genome-wide association study of unexplained chronic ALT elevation as a proxy for nonalcoholic fatty liver disease with histological and radiological validation. Nat Genet 54(6):761–771. 10.1038/s41588-022-01078-z 35654975 10.1038/s41588-022-01078-z PMC10024253

[46] Koscielny G, An P, Carvalho-Silva D et al (2017) Open Targets: a platform for therapeutic target identification and validation. Nucleic Acids Res 45(D1):D985–D994. 10.1093/nar/gkw1055 27899665 10.1093/nar/gkw1055 PMC5210543

[47] MacArthur J, Bowler E, Cerezo M et al (2017) The new NHGRI-EBI Catalog of published genome-wide association studies (GWAS Catalog). Nucleic Acids Res 45(D1):D896–D901. 10.1093/nar/gkw1133 27899670 10.1093/nar/gkw1133 PMC5210590

