# Supplementary material for: Implicating type 2 diabetes effector genes in relevant metabolic cellular models using promoter-focused Capture-C
Source: Diabetologia. 2024 Sep 6;67(12):2740–53. doi: 10.1007/s00125-024-06261-x (PMC11604697; doi:10.1007/s00125-024-06261-x)
Supplement: Supplementary file 1 — ESM (PDF 478 KB) [file 125_2024_6261_MOESM1_ESM.pdf]

Electronic Supplementary Methods

Cell Culture

HepG2 Cells ([HEPG2 (ATCC, HB8065TM)) were maintained in Eagle's Minimum Essential Medium (ATCC, 30 2003) supplemented with 10% heat inactivated fetal bovine serum (Gibco,16000044) and 1X Antibiotic-Antimycotic (Gibco, 15240096) at standard tissue culture conditions; 37C and 5% CO2.

SGBS cells: Differentiation was performed as previously described [1]. Differentiation of SGBS began by washing cells with phosphate buffered saline (PBS) three times and changing to a serum- and albumin-free differentiation medium (DMEM/F12 supplemented with 2 μmol/l rosiglitazone, 25 nmol/l dexamethasone, 0.5 mmol/l methylisobuthylxantine, 0.1 μmol/l cortisol, 0.01 mg/ml transferrin, 0.2 nmol/l triiodotyronin, and 20 nmol/l human insulin). After 4 days, the differentiation medium was changed, and cells were further cultured in DMEM/F12 supplemented with 0.1 μmol/l cortisol, 0.01 mg/ml transferrin, 0.2 nmol/l triiodotyronin, and 20 nmol/l human insulin. Successful differentiation was assessed by the presence of lipid droplets.

ATAC-seq

A total of 50,000 to 100,000 sorted cells were centrifuged at 550g for 5 min at 4°C. The cell pellet was washed with cold PBS and resuspended in 50 μL cold lysis buffer (10 mM Tris-HCl, pH 7.4, 10 mM NaCl, 3 mM MgCl2, 0.1% NP-40/IGEPAL CA-630) and immediately centrifuged at 550g for 10 min at 4°C. Nuclei were resuspended in the Nextera transposition reaction mix (25 ul 2x TD Buffer, 2.5 uL Nextera Tn5 transposase (Illumina, USA Cat #FC-121-1030), and 22.5 ul nuclease free H_2O) on ice, then incubated for 45 min at 37°C. The tagmented DNA was then purified using the Qiagen MinElute kit eluted with 10.5 μL Elution Buffer (EB). Ten microliters of purified tagmented DNA was PCR amplified using Nextera primers for 12 cycles to

generate each library. PCR reaction was subsequently cleaned up using 1.5x AMPureXP beads (Agencourt, USA), and concentrations were measured by Qubit. Libraries were paired-end sequenced on the Illumina NovaSeq 6000 (51 bp read length).

ATAC-seq peaks from libraries were called following the ENCODE ATAC-seq pipeline (https://www.encodeproject.org/atac-seq/). Briefly, pair-end reads from three biological replicates for each cell type were aligned to hg38 genome using bowtie2, and duplicate reads were removed from the alignment. Narrow peaks were called independently for each replicate using macs2 (-p 0.01 --nomodel --shift -75 --extsize 150 -B --SPMR --keep-dup all --call-summits) and ENCODE blacklist regions were removed from peaks in individual replicates. The reproducible peak set (ATAC-seq peaks called by MACS peaks called with q-value < 0.01) were used as the consensus open chromatin regions (OCRs) for each cell type.

The resulting consensus peak sets recovered an average of approximately ~57% of the input peaks **(Supplementary Figure 1)**. Using peaks found in >50% replicates balances reproducibility without being overly restrictive [2].

Promoter Focused Capture C

For each library, 10^7 fixed cells were thawed at 37ºC, followed by centrifugation at RT for 5 mins at 1845 xg. The cell pellet was resuspended in 1 mL of dH2O supplemented with 5 uL 200X protease inhibitor cocktail, incubated on ice for 10 mins, then centrifuged. Cell pellet was resuspended to a total volume of 650 uL in dH2O. 50 uL of cell suspension was set aside for pre-digestion QC, and the remaining sample was divided into 3 tubes. Both pre-digestion controls and samples underwent a pre-digestion incubation in a Thermomixer (BenchMark) with the addition of 0.3%SDS, 1x NEB DpnII restriction buffer, and dH2O for 1hr at 37ºC shaking at 1,000 rpm. A 1.7% solution of Triton X-100 was added to each tube and shaking was continued for another hour. After pre-digestion incubation, 10 ul of DpnII (NEB, 50 U/µL) was added to each sample tube only and continued shaking along with pre-digestion control until the end of

the day. An additional 10 µL of DpnII was added to each digestion reaction and digested overnight. The next day, a further 10 µL DpnII was added and continue shaking for another 2-3 hours. 100 uL of each digestion reaction was then removed, pooled into one 1.5 mL tube, and set aside for digestion efficiency QC. The remaining samples were heat inactivated incubated at 1000 rpm in a MultiTherm for 20 min at 65°C to inactivate the DpnII, and cooled on ice for 20 additional minutes. Digested samples were ligated with 8 uL of T4 DNA ligase (HC ThermoFisher, 30 U/µL) and 1X ligase buffer at 1,000 rpm overnight at 16°C in a MultiTherm. The next day, an additional 2 µL of T4 DNA ligase was spiked in to each sample and incubated for another few hours. The ligated samples were then de-crosslinked overnight at 65°C with Proteinase K (20 mg/mL, Denville Scientific) along with pre-digestion and digestion control. The following morning, both controls and ligated samples were incubated for 30 min at 37°C with RNase A (Millipore), followed by phenol/chloroform extraction, ethanol precipitation at -20°C, the 3C libraries were centrifuged at 1000 xg for 45 min at 4°C to pellet the samples. The controls were centrifuged at 1845 xg. The pellets were resuspended in 70% ethanol and centrifuged as described above. The pellets of 3C libraries and controls were resuspended in 300uL and 20µL dH2O, respectively, and stored at −20°C. Sample concentrations were measured by Qubit. Digestion and ligation efficiencies were assessed by gel electrophoresis on a 0.9% agarose gel and by quantitative PCR (SYBR green, Thermo Fisher). The HepG2 libraries were prepared following the Arima Promoter Capture HiC protocol, using two restriction enzymes (targeting ^GATC and G^ANTC).

For Promoter focused Capture C, isolated DNA from 3C libraries was quantified using a Qubit fluorometer (Life technologies), and 10 µg of each library was sheared in dH2O using a QSonica Q800R to an average fragment size of 350bp [26,28–31]. QSonica settings used were 60% amplitude, 30s on, 30s off, 2 min intervals, for a total of 5 intervals at 4 °C. After shearing, DNA was purified using AMPureXP beads (Agencourt). DNA size was assessed on a

Bioanalyzer 2100 using a DNA 1000 Chip (Agilent) and DNA concentration was checked via Qubit. SureSelect XT library prep kits (Agilent) were used to repair DNA ends and for adaptor ligation following the manufacturer protocol. Excess adaptors were removed using AMPureXP beads. Size and concentration were checked by Bioanalyzer using a DNA 1000 Chip and by Qubit fluorometer before hybridization. One microgram of adaptor-ligated library was used as input for the SureSelect XT capture kit using manufacturer protocol and our custom-designed 41K promoter Capture-C library. The quantity and quality of the captured library was assessed by Bioanalyzer using a high sensitivity DNA Chip and by Qubit fluorometer. SureSelect XT libraries were then paired-end sequenced either the Illumina HiSeq 4000 or on Illumina NovaSeq 6000 platform (51 bp read length). EndoC-BH1, SGBS_undiff, and SGBS_diff libraries were generated in triplicate, while HepG2 were generated in duplicate.

Our capture baits were designed using Agilent SureSelect RNA probes targeting both ends of the *DpnII* restriction fragments containing promoters for coding mRNA, non-coding RNA, antisense RNA, snRNA, miRNA, snoRNA, and lincRNA transcripts (UCSC lincRNA transcripts and sno/miRNA under hg19 assembly) totaling 36,691 RNA baited fragments through the genome[3]. Sequencing data were analyzed as described before except using hg38 as the genome reference and HiCUP 0.7.4 [3], with bowtie2 as the aligner and hg38 as the reference genome. Significant promoter interactions at 1-DpnII fragment resolution were called using CHiCAGO (v1.1.8) [4] with default parameters except for binsize set to 2500. Significant interactions at 4-DpnII fragment resolution were also called using CHiCAGO with artificial.baitmap and .rmap files in which DpnII fragments were concatenated *in silico* into 4 consecutive fragments using default parameters except for removeAdjacent set to False. The significant interactions (CHiCAGO score > 5) from both 1-fragment and 4-fragment resolutions were exported in .ibed format.

Hi-C library preparation and analysis

Hi-C library preparation on two replicates of Endo-C BH1 cells was performed using the Arima-HiC kit (Arima Genomics; cat# A410030), according to the manufacturer's protocols. Briefly, cells were crosslinked using formaldehyde. Crosslinked cells were then subject to the Arima-HiC protocol, which utilizes multiple restriction enzymes (targeting ^GATC and G^ANTC) to digest chromatin. Arima-HiC sequencing libraries were prepared by first shearing purified proximally-ligated DNA and then size-selecting 200–600 bp DNA fragments using AmpureXP beads (Beckman Coulter; cat# A63882). The size-selected fragments were then enriched using Enrichment Beads (provided in the Arima-HiC kit), and then converted into Illumina-compatible sequencing libraries with the Swift Accel-NGS 2S Plus DNA Library Kit (Swift, 21024) and Swift 2S Indexing Kit (Swift, 26148). The purified, PCR-amplified DNA underwent standard QC (qPCR, Bioanalyzer, and KAPA Library Quantification [Roche, KK4824]) and was sequenced with unique single indexes on the Illumina NovaSeq 6000 Sequencing System at 2x101 base pairs.

HiC data was processed as described before [5]. Fastq files were aligned to hg38 as the genome reference and HiCUP 0.7.4 [3], with bowtie2 as the aligner and hg38 as the reference genome. The alignment files (bam) were parsed to.pairs format using pairtools v 0.3.0 and pairix v0.3.7, and eventually converted to pre-binned Hi-C matrix in.cool format by cooler v 0.8.10 with multiple resolutions. Matrices were subsequently merged and normalized with the ICE method [6]. Finally, for each cell type, significant intra-chromosomal interaction loops were determined under multiple resolutions (1, 2 and 4kb) using the Hi-C loop callers mustache v1.0.1 (q-value < 0.1) [7] and Fit-Hi-C2 [8] v2.0.7 (FDR < 1 × 10−6) on merged replicates matrix. The called loops were merged between both callers to represent cell type loops at each resolution.

RNA-seq

Briefly, the purified first-strand cDNA was amplified into RNA-seq libraries using SeqAmp DNA Polymerase and the Forward and the Reverse PCR Primers from the Illumina Indexing Primer

Set HT for Illumina. Quality and quantity of the libraries was assessed using the Agilent 2100 Bioanalyzer system and Qubit fluorometer (Life Technologies). Sequencing of the finalized libraries was performed on the NovaSeq 6000 platform at the CHOP Center for Spatial and Functional Genomics. Fastq files were aligned to hg38 using the STAR aligner (version 2.7.9a). Read counts for genes were determined using HTSeq with gencode v30 as the gene reference set. TPM values were calculated using the effective gene length and average across replicates.

Partitioned LD Score regression

Partitioned heritability LD Score Regression (v1.0.0) [9] was used to identify enrichment of GWAS summary statistics among open accessible regions identified in each cell type. The baseline analysis was performed using LDSCORE data (https://data.broadinstitute.org/alkesgroup/LDSCORE) with LD scores, regression weights, and allele frequencies from 1000G Phase1 and summary statistics from the European type 2 diabetes GWAS [10]. We generated partitioned LD score regression annotations for each cell type using the coordinates of the all-promoter OCR + promoter-interacting OCR. Finally, the cell-type-specific partitioned LD scores were compared to baseline LD scores to measure enrichment in each cell type independently.

Epigenome Roadmap enrichment:

Liftover segment files epigenome roadmap data for 15 state emission files for pancreas (Pancreas_E098_15_coreMarks_hg38lift_segments.bed.gz), liver (Liver_E066_15_coreMarks_hg38lift_segments.bed.gz), and adipose (Adipocyte_E063_15_coreMarks_hg38lift_segments.bed.gz) annotations were retrieved from the webserver on Dec 22, 2021 (https://egg2.wustl.edu/roadmap/web_portal/). We calculated enrichment for the chromHMM to matching cell line promoter interacting region as the ratio of the size of nucleotides within the intersect of the promoter interacting regions and emission to the epigenome roadmap, promoter interacting regions, and genome size.

GWAS data integration

We curated the lead SNPs from the two recent type 2 diabetes GWAS studies[10, 11] and identified proxies in LD with significant type 2 diabetes loci using LDLinkR [12] with R^2>0.8 in matching ancestry. For the recent trans ancestral GWAS sentinel SNPs, proxies in LD with the sentinel SNP in at least two ancestries were selected. We converted the hg19 SNP coordinates provided by LD link to hg38 using liftOver. The set of type 2 diabetes sentinel and proxy SNPs were intersected with the set of OCR annotated to promoter regions (-1500/+500bp of TSS) and OCR overlapping promoter interacting regions identified by Capture C. Genomic coordinate overlaps were identified using the R package GenomicRanges [13] (ver 1.42) with the human genome reference hg38.

Transcription Factor analysis

Transcription factor binding site motifs overlapping with proxies implicated in by V2G mapping analysis were identified using the R package motifbreakR (v2.0.0)[69] using the JASPAR2020 database as the reference set of position weight matrices[70]. For comparisons to ENCODE Data, TF-ChIP peak coordinates in hg38 were retrieved using curl. We used Genomic Ranges findOverlaps with the set of V2G implicated SNPs.

EndoC-BH1 siRNA transfection

For knockdown experiments, EndoC-BH1 cells were transfected with siRNA using DharmaFECT1 transfection reagent (Dharmacon, Catalog # T-2001-01) following the Manufacturer's instructions. Dharmacon ON-TARGETplus SmartPool (FXR2 Catalog # L-011955-00-0005, SMCO4 Catalog # L-020414-02-0005, and Non-Targeting Catalog # D-001810-10-05) were used at 37.5 nM for SMCO4 and 50 nM for Non-Targeting and *FXR2*. First, 24 hours prior to siRNA treatment, cells were seeded into a 12-well β-coated plate at 125,000

cells per well. The next day, the cells were treated with the appropriate siRNA (Non-Targeting control, *FXR2*, or *SMCO4*) with 1 mL of 5µM siRNA in 1x siRNA buffer (Dharmacon, Catalog # B-002000-UB-100) was dispensed to each well. The cells following siRNA transfection were then used for glucose stimulated insulin secretion assays.

Human Insulin ELISA

Initially, 25 µL of the calibrators, non-targeting controls, and test samples (*FXR2* siRNA, *SMCO4* siRNA) were dispensed into the appropriate wells in triplicate. 100 µL of enzyme conjugate 1x solution was introduced into each well, and the plate was incubated on a shaker at 700 rpm for 1 hour at room temperature. Following this, the reaction volume was discarded by inverting the microplate over the sink, and the wells were subjected to five washes with 1x wash buffer, and excess liquid with absorbent paper. Next, 200 µL of Substrate TMB was added to each well and incubated for 15 minutes on the benchtop. Finally, 50 µL of Stop Solution was added to each well, and the plate was briefly placed on a shaker to ensure proper mixing. Optical density readings were then obtained at 450 nM using the Molecular Devices SpectraMax ID5.

Quantitative RT-PCR

First, for the reaction 10 µL Taqman Fast Advanced Master Mix, 1 µL target gene (*FXR2* or *SMCO4*), 1 µL Endogenous Control Gene (actin beta), 6 µL nuclease-free water, and 2 µL cDNA (200 ng/µL) or 2 µL of nuclease-free water for No Template Control. Next, 20 µL of the reaction was pipetted into a 96-well standard (0.2-mL) plate in triplicate for each treatment including a No Template Control (NTC). The reaction was run for 1 cycle of 50°C for 2 minutes, 95°C for 20 seconds, followed by 40 cycles, 95°C for 3 seconds then 60°C for 3 minutes.

References:

[1] Tews D, Brenner RE, Siebert R, Debatin KM, Fischer-Posovszky P, Wabitsch M (2022) 20 Years with SGBS cells - a versatile in vitro model of human adipocyte biology. Int J Obes (Lond) 46(11): 1939-1947. 10.1038/s41366-022-01199-9

[2] Yang Y, Fear J, Hu J, et al. (2014) Leveraging biological replicates to improve analysis in ChIP-seq experiments. Comput Struct Biotechnol J 9: e201401002. 10.5936/csbj.201401002
[3] Chesi A, Wagley Y, Johnson ME, et al. (2019) Genome-scale Capture C promoter interactions implicate effector genes at GWAS loci for bone mineral density. Nat Commun 10(1): 1260. 10.1038/s41467-019-09302-x
[4] Cairns J, Freire-Pritchett P, Wingett SW, et al. (2016) CHiCAGO: robust detection of DNA looping interactions in Capture Hi-C data. Genome Biol 17(1): 127. 10.1186/s13059-016-0992-2
[5] Su C, Gao L, May CL, et al. (2022) 3D chromatin maps of the human pancreas reveal lineage-specific regulatory architecture of T2D risk. Cell Metab 34(9): 1394-1409 e1394. 10.1016/j.cmet.2022.08.014
[6] Imakaev M, Fudenberg G, McCord RP, et al. (2012) Iterative correction of Hi-C data reveals hallmarks of chromosome organization. Nat Methods 9(10): 999-1003. 10.1038/nmeth.2148
[7] Roayaei Ardakany A, Gezer HT, Lonardi S, Ay F (2020) Mustache: multi-scale detection of chromatin loops from Hi-C and Micro-C maps using scale-space representation. Genome Biol 21(1): 256. 10.1186/s13059-020-02167-0
[8] Kaul A, Bhattacharyya S, Ay F (2020) Identifying statistically significant chromatin contacts from Hi-C data with FitHiC2. Nat Protoc 15(3): 991-1012. 10.1038/s41596-019-0273-0
[9] Finucane HK, Bulik-Sullivan B, Gusev A, et al. (2015) Partitioning heritability by functional annotation using genome-wide association summary statistics. Nat Genet 47(11): 1228-1235. 10.1038/ng.3404
[10] Mahajan A, Taliun D, Thurner M, et al. (2018) Fine-mapping type 2 diabetes loci to single-variant resolution using high-density imputation and islet-specific epigenome maps. Nat Genet 50(11): 1505-1513. 10.1038/s41588-018-0241-6
[11] Vujkovic M, Keaton JM, Lynch JA, et al. (2020) Discovery of 318 new risk loci for type 2 diabetes and related vascular outcomes among 1.4 million participants in a multi-ancestry meta-analysis. Nat Genet 52(7): 680-691. 10.1038/s41588-020-0637-y
[12] Myers TA, Chanock SJ, Machiela MJ (2020) LDlinkR: An R Package for Rapidly Calculating Linkage Disequilibrium Statistics in Diverse Populations. Front Genet 11: 157. 10.3389/fgene.2020.00157
[13] Lawrence M, Huber W, Pages H, et al. (2013) Software for computing and annotating genomic ranges. PLoS Comput Biol 9(8): e1003118. 10.1371/journal.pcbi.1003118

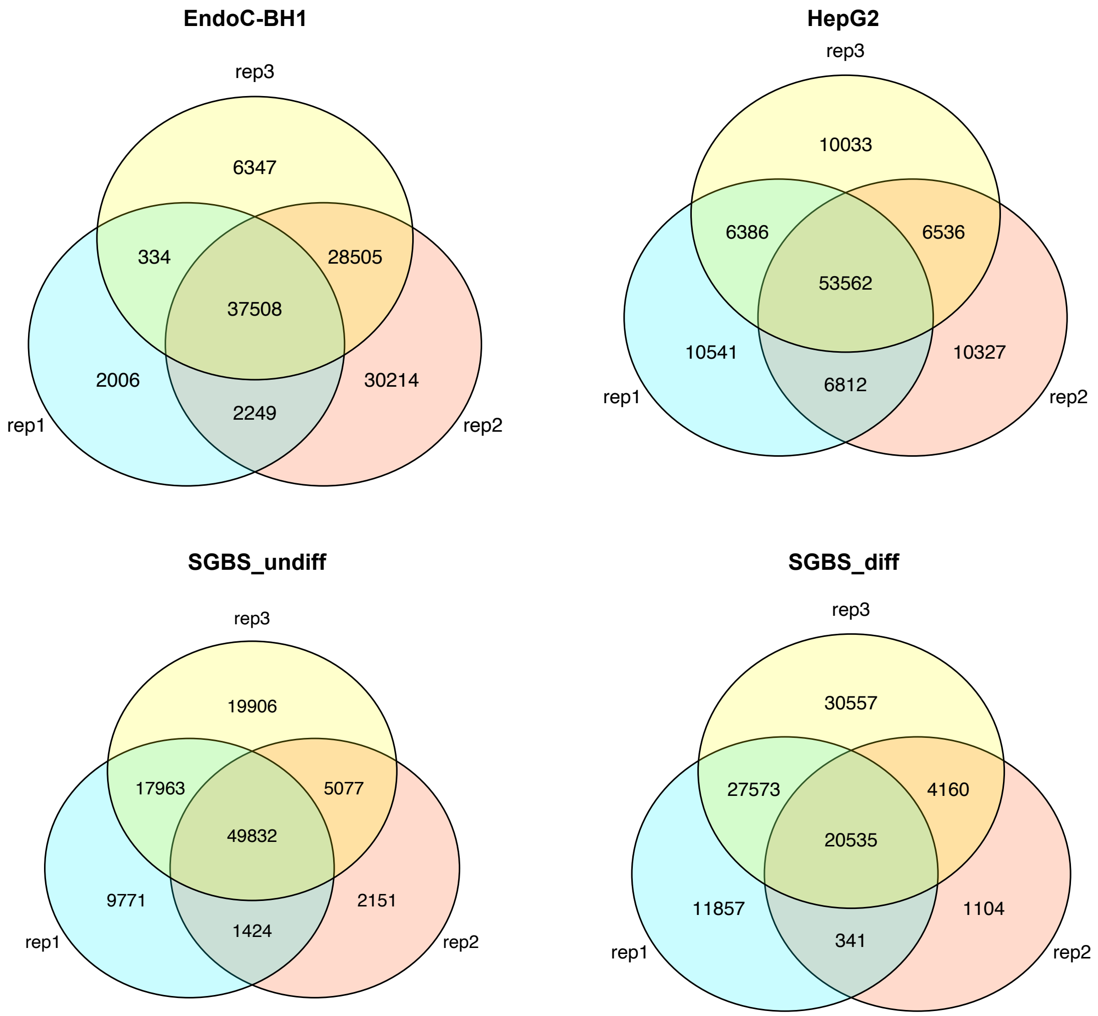

ESM Fig. 1: Venn Diagrams depicting the overlap between three replicates for EndoC-BH1, HepG2, SGBS_undiff, and SGBS_diff cells. The number of OCRs in each category are shown.

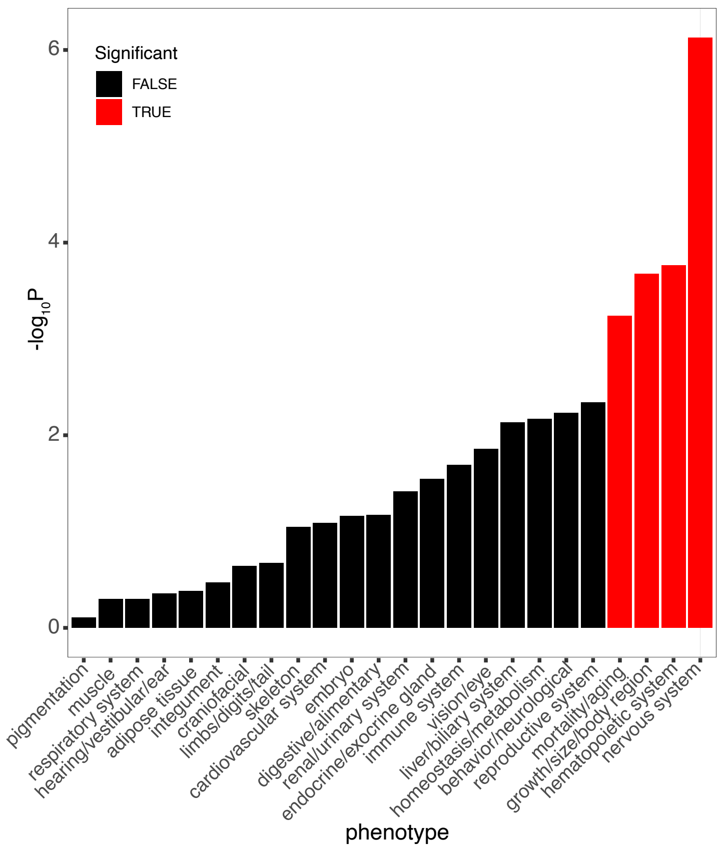

ESM Fig. 2: Comparison of implicated genes to the international mouse phenotyping consortium. For each transcription factor we calculated the fraction of motifs that overlapped with a ChiP peak in at least one tissue type. Red bars are traits that are significant (FDR < 0.05).

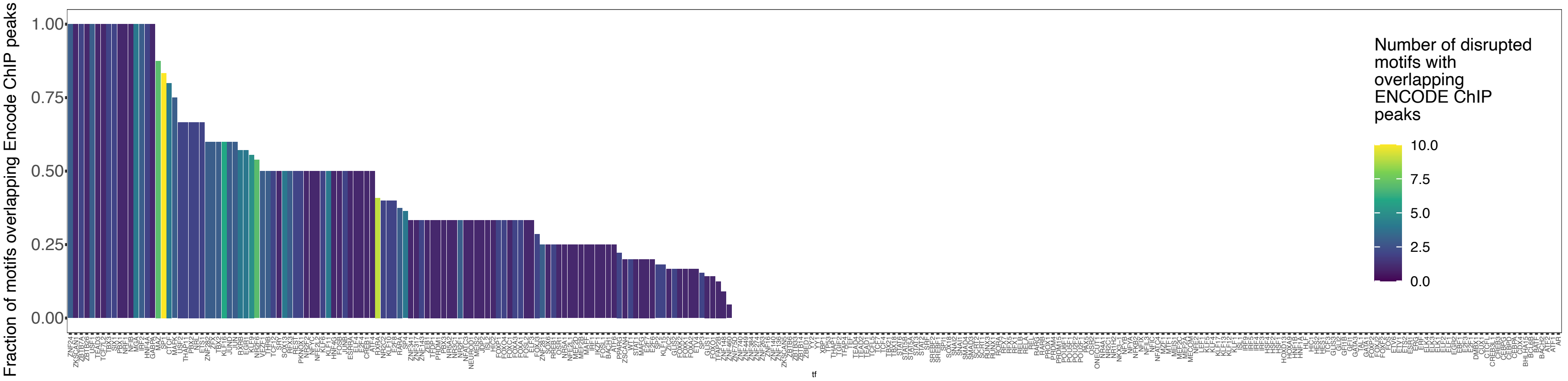

ESM Fig. 3: Comparison of implicated gene. For each transcription factor we calculated the fraction of motifs that overlapped with a ChiP peak in at least one tissue type. The y-axis indicates the fraction of motifs with an overlapping with a ChiP-seq peak. Color indicates the absolute number of motifs overlapping.

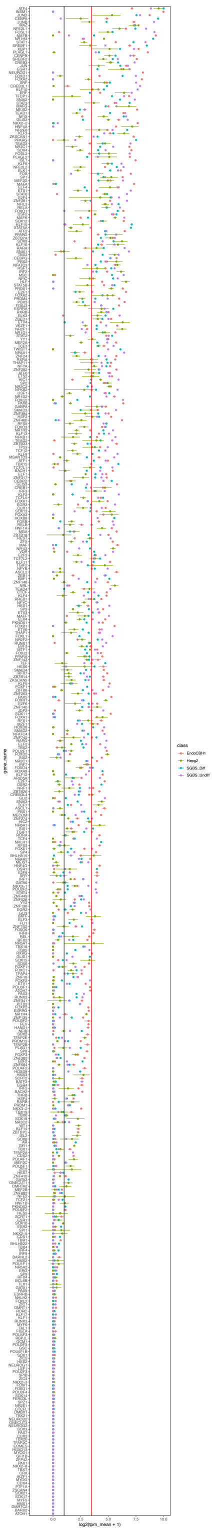

ESM Fig. 4: Expression (TPM values) of transcription factors implicated by motif analyses. Lines correspond to TPM = 1 (black) and TPM = 10 (red).

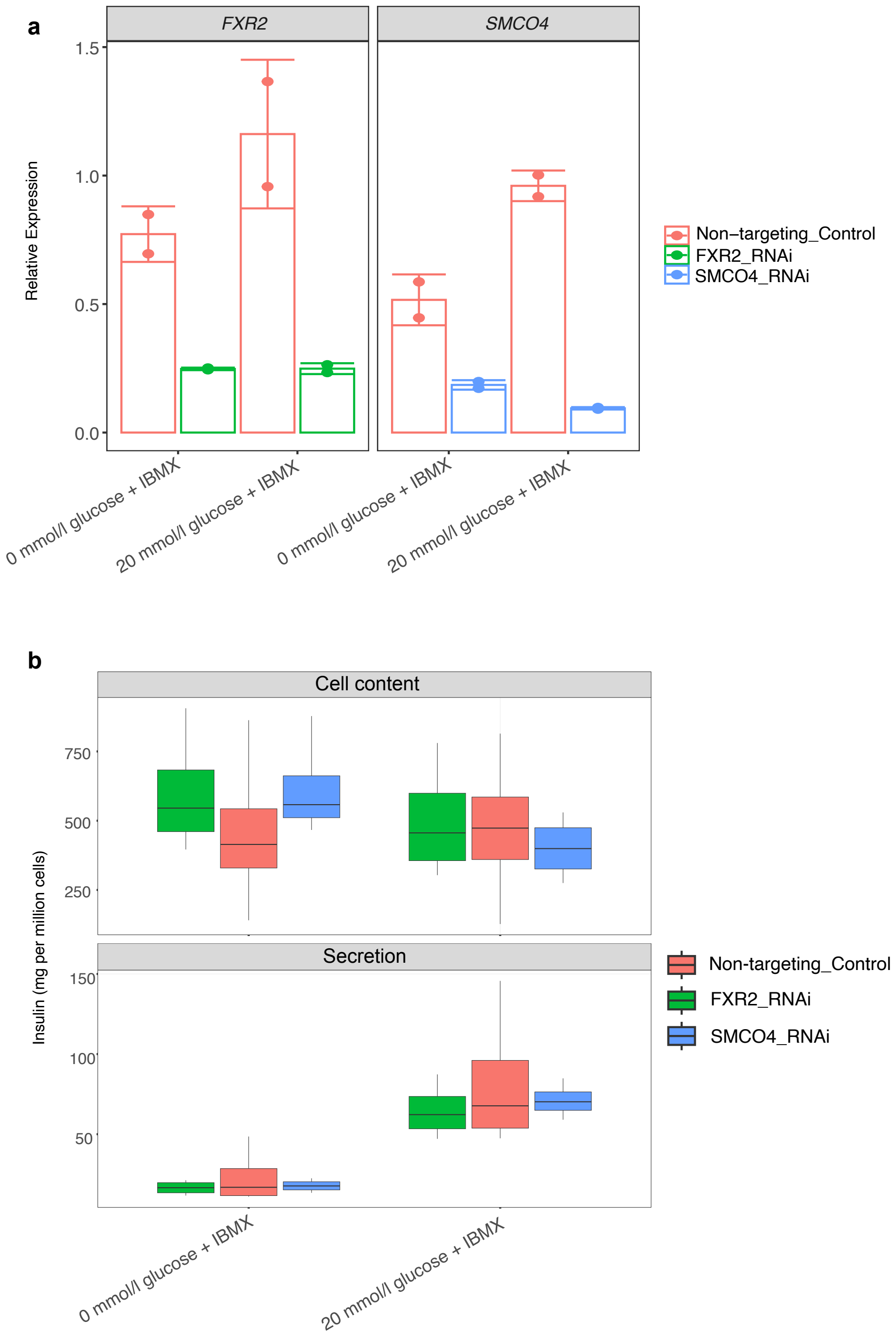

ESM Fig. 5: No effect on IBMX mediated secretion observed with knockdown of *SMCO4* or *FXR2* expression. (A) qPCR validation of *SMCO4* and *FXR2* knockdown at either 0mM or 20mM glucose performed with IBMX (n=2). Measurements are adjusted to be comparable to basal non-targeting controls (Fig 4D). (B) Insulin secretion assay with IBMX, no significant differences were observed. The mean of six technical replicates was taken for four biological replicates. Paired two tailed t-tests were used to assess statistical significance across the four biological replicates. * indicates $P < 0.05$.
